# Chemical analysis of low grade gold from mine tailings after size fractionation and acid digestion using reverse aqua regia

**DOI:** 10.1038/s41598-025-94515-y

**Published:** 2025-03-24

**Authors:** Kedibone Nicholine Mashale, James Sehata, Napo Godwill Ntsasa, Luke Chimuka, James Tshilongo

**Affiliations:** 1 Analytical Chemistry Division, Mintek, 200 Malibongwe Drive, Praegville, 2194 South Africa; 2 School of Chemistry, University of the Witwatersrand, Johannesburg, 2050 South Africa

**Keywords:** Gold tailings, Acid digestion, Measurement uncertainty, Fire assay, Environmental sciences, Chemistry

## Abstract

The growing interest in reprocessing mine tailings for gold recovery requires a suitable quantification method that is accurate, rapid, and not harsh to the environment. Acid digestion is often used to determination of gold; however, it often faces the challenge of incomplete digestion due to the presence of minerals such as quartz, and homogeneity is compromised due to small sample masses, which can result in low bias. This study investigated a shorter acid digestion method employing reverse aqua regia, both in the presence and absence of hydrofluoric acid. Before digestion, the sample was subjected to gold depot analysis, which showed that 78% was free-milling gold and that only 0.8% was associated with pyrite, increasing the chances of accurate quantifications. Furthermore, the size screening test showed that most of the gold could be recovered on the − 38 μm screen. This proposed method provided good linearity (5–100 µg. L^− 1^) and low detection limits (0.139–0.183 µg.kg^− 1^). The concentrations obtained by the acid digestion was 0.258 g.t^− 1^ with the recoveries ranging between 80% and 82%, which fit the criteria set. The method also worked well for the certified reference materials (CRM), AMIS 610 (accurate value = 0.068 g.t^− 1^) and AMIS 646 (accurate value = 0.166 g.t^− 1^), which are of a similar matrix and are also lower in grade compared to the sample. The method was also evaluated for uncertainty (± value) using the bottom-up approach, and the expanded uncertainty (k = 2) was reported to be 0.258 ± 0.092 g.t^− 1^, which was comparable to that offered by the fire assay with the ICP‒OES finish, which was 0.28 ± 0.10 g.t^− 1^. This implies that the acid digestion method is suitable for quantifying gold from mine tailings without large uncertainties.

## Introduction

Recently, there has been interest in the reprocessing of mine tailings for the extraction of any valuable minerals lost due to inefficient processes. This is a great initiative that benefits the environment and mining companies in that less energy is needed, as the material has already been removed from the earth’s crust, decreases the number of mine-related deaths, and has financial benefits^1^. This, however, means that the methods used for quantifying minerals such as gold need to be improved or that new methods need to be developed. This is because, for a typical gold mine tailing sample, the ratio of gold to gangue material is approximately 1:10^2^.

For decades, fire assay (FA) has been used as the conventional method for the quantification of gold from various ores and is usually coupled to gravimetric analysis or instrumental analysis, inductively coupled plasma optical emission spectroscopy (ICP‒OES), inductively coupled plasma‒mass spectrometry (ICP‒MS), atomic absorption spectroscopy (AAS), etc. The challenge with fire assay is that due to the ratio of gold to gangue material, a large sample mass must be used to obtain a prill that can be accurately weighed and is easy to subject to other procedures; additionally, it requires highly skilled assayers and uses lead, which is toxic to humans^3^. Additionally, the amount of the sample used versus the amount of gold that will be determined, is it economically and environmentally beneficial?

For the quantification of gold, various methods exist other than fire assays, namely, acid digestion, direct analysis using instrumental neuron activation analysis (INAA), or laser ablation techniques; however, the grade of the gold is usually a limitation. In terms of acid digestion, which has been studied mostly for medium- to high-grade gold ores and includes the digestion or decomposition of the sample using acids assisted by heat, various challenges have been reported^4^. The challenges include the presence of minerals such as pyrite, quartz, and chromite, which can hinder the digestion process, especially when a large amount of gold is enclosed in those minerals^5^.

Acid digestion usually involves the combination of various acids, such as nitric acid (HNO_3_), hydrochloric acid (HCl), perchloric acid (HClO_4_), sulfuric acid (H_2_SO_4_) and hydrofluoric acid (HF), to achieve synergistic effects^6^. Because of the incomplete digestion of the material due to encapsulation of the gold grains, the digestion time and acid quantities need to be increased, and the possible use of HF decomposes the quartz in the sample and removes it as SiF_4_^6,7^. However, environmentally, it is not beneficial, as it requires more energy. Since tailings have already been processed, the assumption is that it might be easier to digest the tailings with less harsh conditions (acid combinations and time) and still be able to accurately quantify the amount of gold^7^.

In addition to the incomplete acid digestion that occurs, another problem is the homogeneity of the gold in the sample and the relation of the small sample mass to the homogeneity. This is mainly because the grains that contain gold are heterogeneously distributed in the ore, which eventually leads to the formation of gold nuggets^8^, which is often observed in the analysis of replicates. For fire assays, this problem is often overcome by using large sample masses (> 50 g for a tailings sample), which also improves the accuracy and precision of the analysis. In acid digestion, the typical masses used are less than 20 g, some of which are as low as 5 g^6,9,10^. In this case, it is recommended that the majority of the particles in the sample be at least 35 µm. Upon chemical analysis, the level of homogeneity in the sample can be calculated using the homogeneity indicator (Eq. 1), in which the relative standard deviation *(RSD)* is calculated from the fractions and *m* is the mass used. Subsequently, Pauwel’s equation (Eq. 2) can be used to calculate the minimum amount (*M)* of sample required to obtain homogenous results at a certain uncertainty *(UNC)* level^11^ and tolerance level *(K*_*2*_^*’*^*).*

1 $$\:{H}_{E}=\left(RSD\right)\:\times\:\sqrt{m}$$

2 $$\:M=\left(\frac{{K}_{2}^{{\prime\:}}\times\:\%RSD)}{UNC}\right)\times\:m$$

Furthermore, the suitability of acid digestion for the quantification of low gold concentrations can be assessed by the uncertainties that arise from chemical analysis. This would strengthen the quantification if there is a level of confidence attached to the analytical results. Although there are various ways to assess this uncertainty, the bottom-up approach would be more advantageous, as it will show the parameters that contribute to larger uncertainties and will allow for that parameter to be assessed and improved. Therefore, in this study, an acid digestion method that offers shorter digestion times and uses the simplest acid combination was explored and compared to existing fire assays. The comparison was performed based on the uncertainties that each method produces.

## Materials and methods

### Materials

All of the study reagents were of high purity and were not altered. Anhydrous sodium carbonate (Na_2_CO_3_), sodium peroxide (Na_2_O_2_), hydrogen peroxide (H_2_O_2_) and sodium cyanide (NaCN) were purchased from Associated Chemical Enterprises Pty Ltd. (Johannesburg, South Africa). Hydrochloric acid (HCl, 37%), perchloric acid (HClO_4_) and nitric acid (HNO_3_, 65%) were purchased from Sigma Aldrich (Missouri, United States of America). The tuning solution for ICP‒MS analysis was purchased from Thermo Fischer Scientific (Massachusetts, United States of America). For ICP‒OES, the tuning solutions were purchased from Agilent Technologies (California, United States of America). The African Mineral Standards (Modderfontein, South Africa) provided the certified reference materials (CRMs) AMIS 610 and AMIS 646. The gold and base metal stock solutions (1000 mg. L^− 1^ and 10 000 mg. L^− 1^) were purchased from LGC Standards (Middlesex, United Kingdom). All solutions were prepared using Millipore water from a Milli-Q system from Sigma Aldrich (Missouri, United States of America).

### Particle size distribution, homogeneity, and purity analysis

The ground sample was divided into smaller portions using a rotary separator, with 24 portions obtained with an average weight of 1 kg each. A Malvern HydroEV instrument and Mastersizer 3000 software (Malvern Pan Analytical Ltd., Worcestershire, UK) were used to measure the particle size distribution (PSD). Additional screening was performed by physical sieves (1180, 600, 425, 300, 212, 150, 106, 75, 53, 38 μm), starting with a wet sieve to remove the finer fraction (termed − 38 μm). This slurry was then dried in an oven overnight at 60 °C, ground to a powder and passed through a series of sieves. The homogeneity of the fractions was assessed by selecting random fractions and chemically analyzing them through mineralogical, elemental composition and fire analyses. Blank samples from the techniques were analyzed to determine reagent purity.

### X-ray diffraction

The material was analyzed by a backloading preparation process after being pulverized in a swing mill to -50 μm. The diffractogram was produced using a Malvern Panalytical Aeris diffractometer (Malvern Pan Analytical Ltd., Worcestershire, United Kingdom) equipped with a PIXcel detector, fixed slits, and Fe-filtered Co-K radiation. The diffractometer has a scanning range of 5–80° and a step scan of 0.022° with a measurement time of 48 s. PAN-ICSD and X’Pert Highscore plus software were used to identify the phases. Using the Rietveld method, the relative phase quantities (wt%) were calculated, with the method detection limit specified as 0.5 wt%.

### Determination of the head grade

The fire assay with lead collection was carried out as outlined in literature^12^. The composition of the flux used was NaCO_3_ (39.14%), fused borax (21.50%), reducing agent (2.08%), paraffin (0.01%), silica (12.75%), litharge (21.50%), and a litharge: reducing agent ratio of 10. The prill obtained was digested with 3 mL of 65% HNO_3_ and then 9 mL of 37% HCl and transferred into a 25 mL volumetric flask with 10 mg. L^− 1^ scandium internal standard. The gold content of this solution was analyzed by ICP‒MS.

### Gold deportment study

Due to the low-grade nature of the gold in the tailings sample, the deportation of gold had to be analyzed using chemical methods, which are 3-stage diagnostic leaches. The leaching involved leaching the sample with cyanide using 10 g.t^− 1^ NaCN for 24 h on a rolling bench (a), a second cyanide leaching with carbon in leach (CIL) (b), a leach using 2.5 M HCl (c), a leaching step with 27% HNO_3_ (d), and roasting at 850 °C for 1 h (e). The gold content obtained from the filtrates and residues was used to calculate the deposition of gold on various minerals, such as quartz, pyrite, and calcite, as shown in Table 1:

**Table 1:** Equations for calculation of the gold deportment.

Fraction	Calculation
Cyanide soluble	Head-a
Preg-robbed	a-b
HCl digestible minerals	b-c
HNO_3_ digestible minerals	c-d
Carbonaceous matter	d-e

### Acid digestion

A 1 g sample was weighed into a Teflon beaker and wetted with deionized water. The acid mixtures used were reverse aqua regia (3HNO_3_: 1HCl) with and without HF addition, totaling two experiments. The ratio of the acids was determined experimentally, and 3:1 was selected as the optimum ratio based on the obtained gold recoveries. After the sample was wetted, 10 mL of the acid mixture was added and allowed to digest on a hot plate (200 °C) for 20 min under reflux. After the initial step, 10 mL of nitric acid (and 10 mL of HF for the experiments with HF addition) was added to further aid the digestion, and the mixture was allowed to warm until dry. The residue was then reconstituted with HCl, and drops of H_2_O_2_ were added to dissolve any remaining residues. The samples were then transferred into 50 mL volumetric flasks containing 10% HCl and 10 mg. L^− 1^ scandium internal standard. The experiments were carried out in triplicate on three samples (VCR, AMIS 610, and AMIS 646), including blanks.

### Instrumental analysis

Analysis of the samples was carried out on a Thermo Fischer Scientific iCap Q instrument with data processing on the Qtegra software (Version: 2.10.3324.62). The samples were introduced into the system using an ASX-520 autosampler, PTFE nebulizer, and a double-pass cyclonic spray chamber. Performance checks and autotuning were carried out daily to ensure the optimum functioning of the instrument. These included mass calibrations, optimizations of the nebulizer flow rate, torch position, and overall system performance. The solution used was a 1 µg. L^− 1^ standard containing Be, Ce, Fe, In, Li, Mg, Pb, and U in 1% HNO_3_. Six-point external calibration curves at 5, 10, 20, 40, 50, and 100 µg. L^− 1^ was prepared from a 1 mg. L^− 1^ working solution, which was prepared from a 1000 mg. L^− 1^ stock solution. The analysis was carried out in both standard and kinetic energy discrimination (KED) modes to compare the two modes of interference correction. To avoid oversaturation of the detector, the samples were diluted using a MicroLab 600 auto dilutor to a final volume of 10 mL.

### Statistical evaluations

All samples and tests were performed in triplicate to obtain a good representation of the analysis and for continued assessment of the homogeneity. Since the sample was split into smaller fractions, the number of fractions to be used to test for homogeneity was calculated following Eq. 3^13^, where N is the number of fractions after splitting the bulk sample:

3 $$\:min=\sqrt[3]{{N}_{fraction\:}\:}and\:max=3\times\:\sqrt[3]{{N}_{fraction}}$$

The uncertainty evaluations were performed with the bottom-up method, which included examining the factors that can affect the quantification of gold using a fire assay and acid digestion with an ICP‒MS finish. The results of these two tests were compared to determine whether their means were significantly different using a paired t test in Microsoft Excel (Microsoft Office Professional Plus 2016). Based on the following model equation (Eq. 4), the factors that were incorporated into the uncertainty analysis were glassware calibration, mass balance, instrument stability, homogeneity, method repeatability, and reproducibility. For repetitive measurements, the standard deviation was used as the standard uncertainty. For mass balance and instrument uncertainty, the uncertainty was obtained from the calibration data^14^ and propagated to give the combined uncertainty at a coverage factor of 2 (k = 2) or 95% confidence interval.

4 $$\:C=\frac{{C}_{0}\times\:V}{W\times\:1000}$$

where *C* is the concentration of the element in the sample, *C*_*0*_ is the element concentration in the sampling concentration, *V* is the volume of the sampling solution and *W* is the mass of the sample. The measurement uncertainty emanating from the different sources was calculated as shown in Table 2.

**Table 2:** Equations used to quantify the measurement uncertainty from various sources (n = number of data sets, s = standard deviation, u = measurement uncertainty).

Uncertainty contributor	Calculation
Weighing (type A)	$\:{u}_{s}=\frac{Expanded\:uncertainty}{coverage\:factor}$ (5)
Glassware (type A)	$\:{u}_{2}=\frac{volume\times\:expansion\:factor\times\:temperature\:difference}{coverage\:factor}$ (6)
Dilution of working standard	$\:{u}_{working\:standar}relative=\sqrt{{\left(\frac{{u}_{stock}}{\left[stock\right]}\right)}^{2}+{\left(\frac{{u}_{v1}}{dispensed\:volume}\right)}^{2}+{\left(\frac{{u}_{v2}}{dispensed\:volume}\right)}^{2}}$ (7)
Repeatability	$\:u=\frac{s}{\sqrt{n}}$ (8)
Reproducibility	$\:u=\frac{s}{\sqrt{n}}$ (from two data sets)
Instrument stability	$\:u=\frac{s}{\sqrt{n}}$ (from two calibration data)

## Results and discussion

### Mineralogical composition

**Fig. 1:**
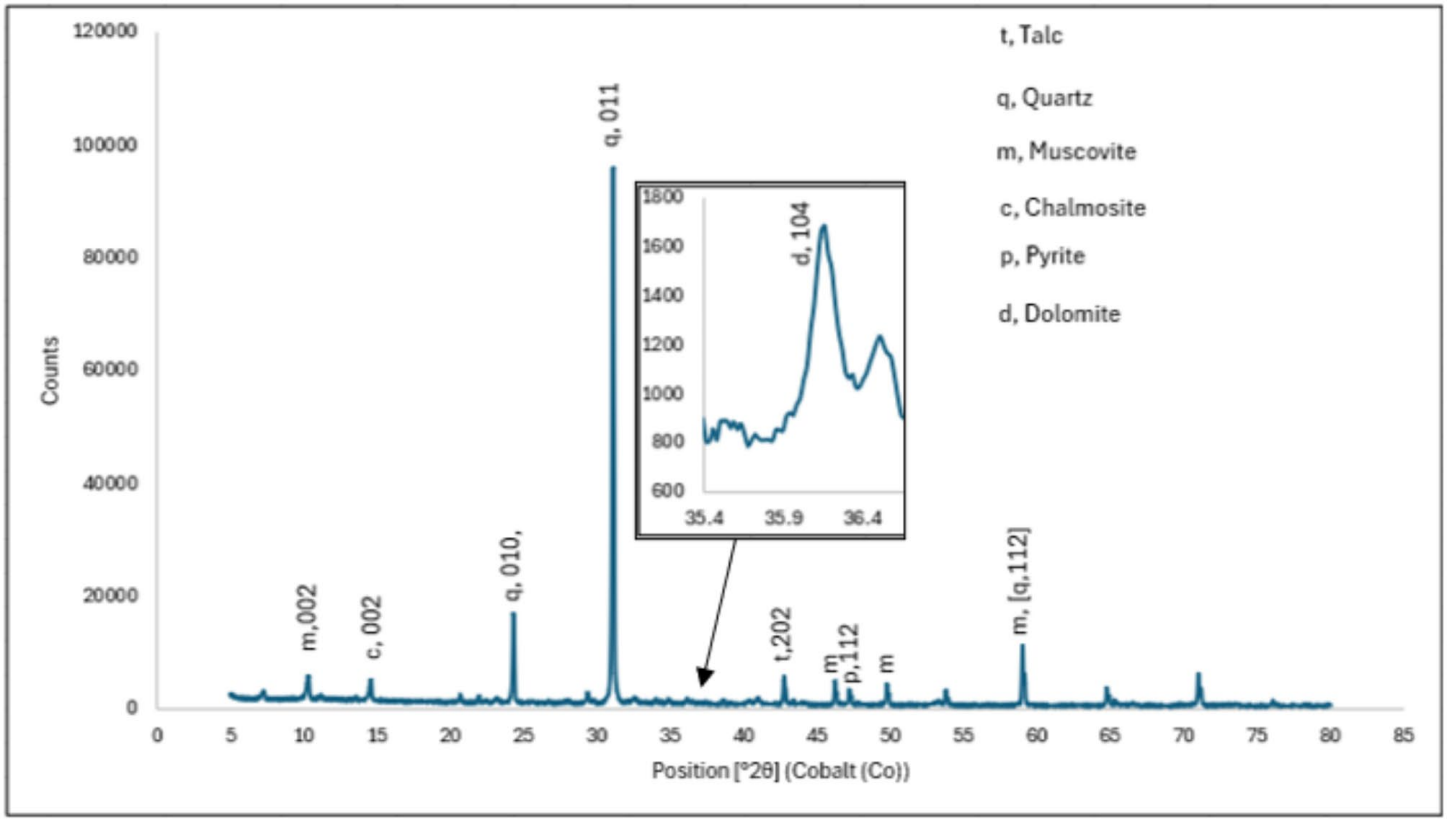
A Diffractogram showing the composition of the tailing material by XRD. The image shows the peaks that were detected and their identification.

The predominant minerals are shown in Table 3; Fig. 1, with quartz (SiO_2_) being the most dominant at approximately 80% by weight and calcite as the minor phase at 0.2% by weight, as expected for the matrix, VCR and GSB^15,16^. The presence of quartz at this percentage indicates the possibility that gold encapsulation may interfere with acid decomposition, while the presence of pyrite (FeS_2_) may interfere with the fire assay and further chemical analysis. Previous studies have shown similar trends in terms of the major and minor minerals detected, including quartz (58–82%), mica (3–10%), and chlorite (3–10%) for the Wits Basin^17^ and quartz, magnetite and magnesioferrite for the East Rand Basin^18^, both of which were quantified by XRD. In terms of mineralogical comparisons of the sample and the CRMs (AMIS 610 and AMIS 646) used in the study, the amounts of quartz were quite similar.

**Table 3:** Mineralogical information of the bulk sample and the certified reference materials in %wt.

Mineral	Ventersdorp contact reef	AMIS 610	AMIS 646
Quartz	78.1	82	62
Pyrite	0.8	n.d	1
Muscovite	11.4	n.d	10
Dolomite	1.4	n.d	n.d
Chamosite	6.9	n.d	n.d
Talc	0.9	5	n.d

The detection limit was classified as 0.5%wt. n.d denotes not detected due to detection limit.

**Fig. 2:**
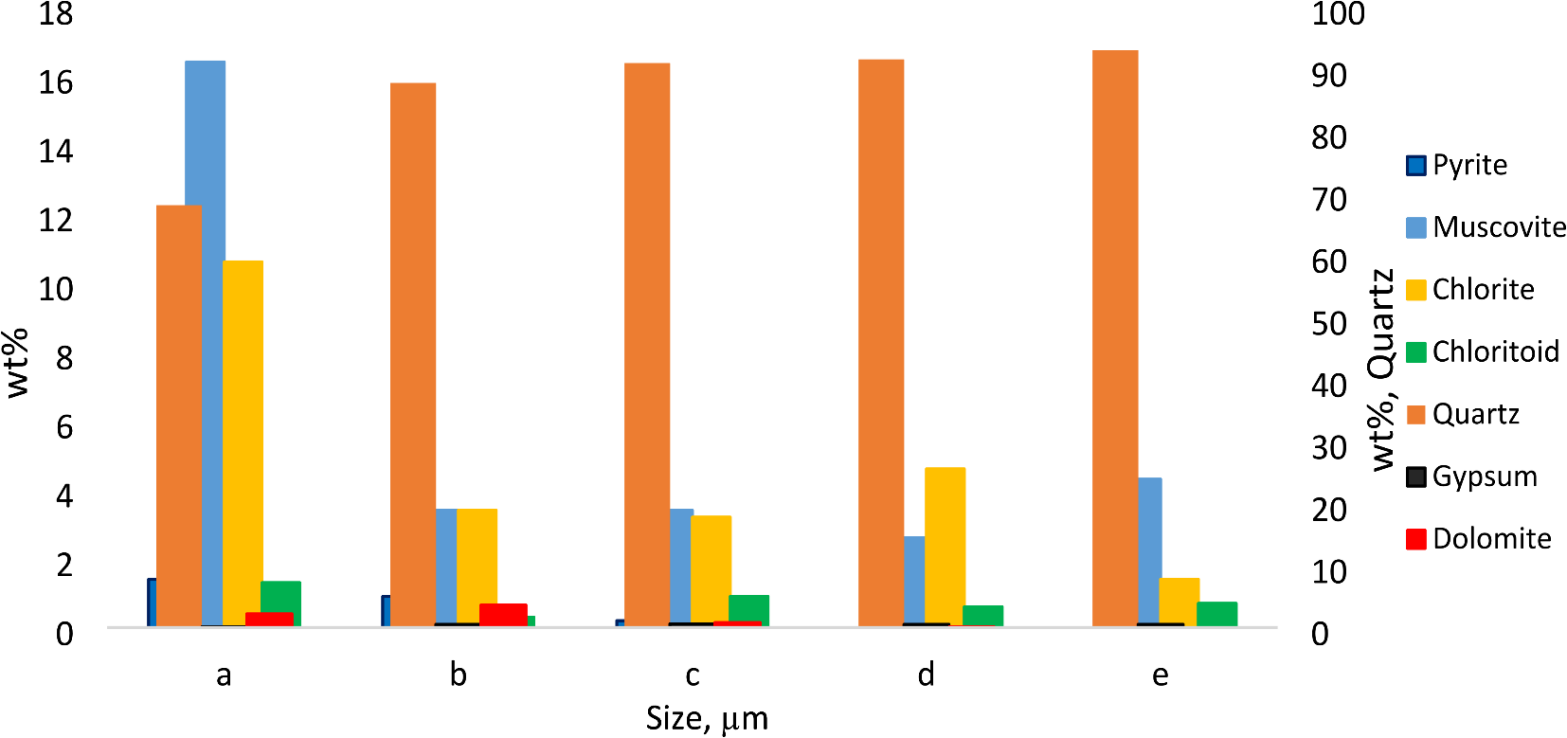
Mineralogical data of the screened fractions (*n* = 3). a= -38 μm, b = + 38 μm, c = + 53 μm, d = + 75 μm and e = + 106 μm.

The assay-by-size analysis showed that quartz was still dominant in each of the screens, with a decreasing trend from the finer screen to the coarser screen (Fig. 2). This type of behavior was also reported in^19^, where the 300 and minus 75 μm screens reported 39% and 15%, respectively. This process is considered to be beneficial because quartz is often known to hinder acid dissolution because it can encapsulate gold, but in this case, the extraction of gold from a finer screen with a low amount of quartz is expected to yield better results.

### Gold depot

A gold deposition study was performed to quantify the gold that is associated with minerals such as pyrite, magnetite, and quartz. Based on these results, an appropriate pretreatment method can be chosen to liberate gold from minerals. In this study, 78% of the gold was recoverable by direct cyanidation, 9.4% was locked in HCl-digestible minerals such as magnetite, chlorites, chamosite, and carbonates, and 3% was locked in minerals such as pyrite. This result correlates with the XRD analysis; chamosite is one of the dominant minerals at 6.9 wt%, while pyrite is one of the lowest at 0.8%. Similar studies have reported percentages of 89%, 83%, and 79–86% for gold recoverable by direct cyanidation and 7.4%, 10.3%, and 13–53% for gold locked in pyrites^19–21^.

**Table 4:** Distribution of gold in various minerals by gold depot.

	Filtrate	Residue	
Au deportment	Au grade, g.t^− 1^	Au grade, g.t^− 1^	Au distribution, %
Cyanide soluble (a)	0.25	0.07	78
Preg-robbed (b)	0.01	0.06	3
HCl digestible minerals (c)	0.03	0.03	9.4
HNO_3_ digestible minerals (d)	0.01	0.02	3
Carbonaceous matter (e)	0.01	0.01	3
Gangue locked	0.05	0.01	15
Total	0.33		100

### Size-based fire assay

**Fig. 3:**
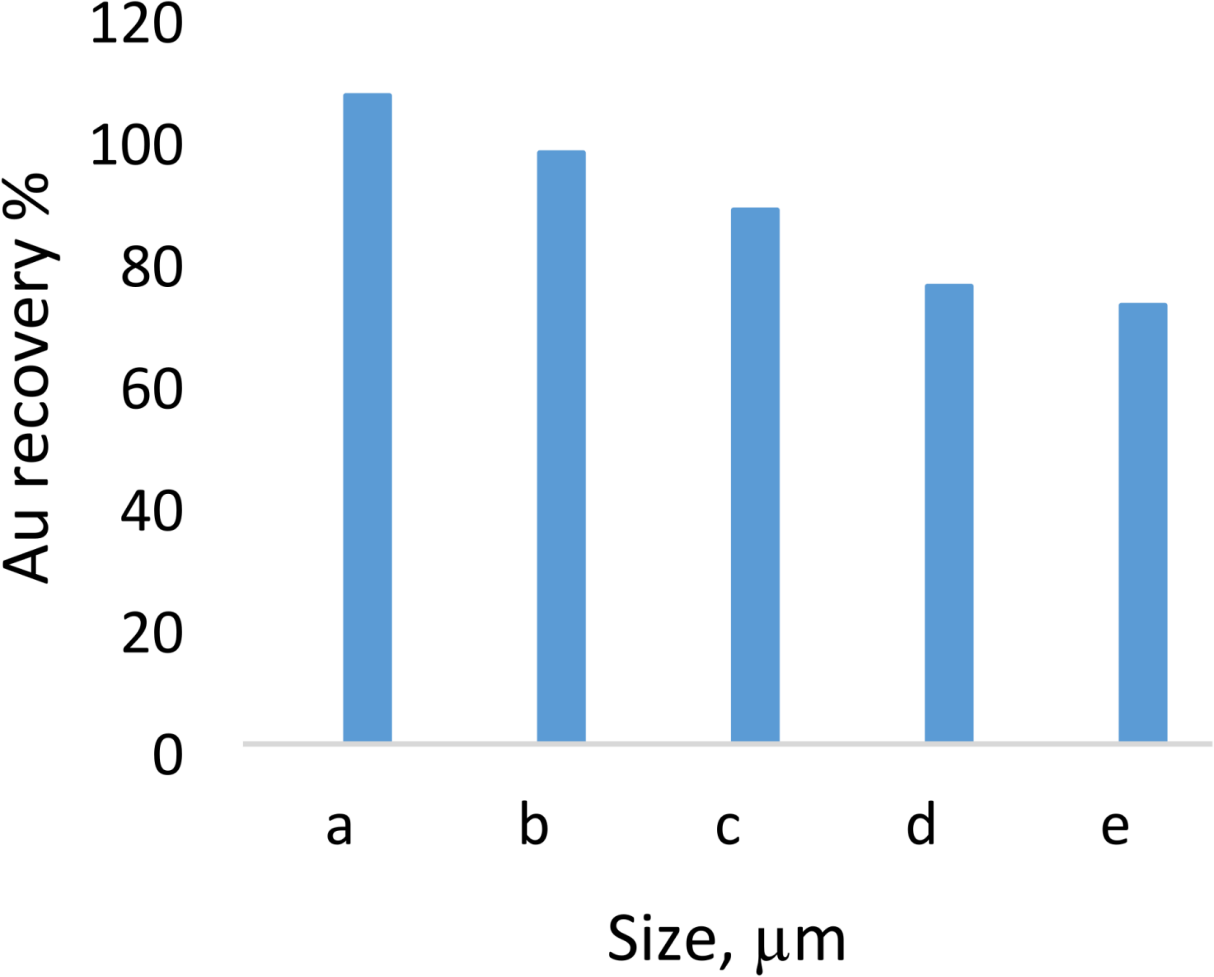
Gold recoveries of the screened fractions (*n* = 3). a= -38 μm, b = + 38 μm, c = + 53 μm, d = + 75 μm and e = + 106 μm.

The recovery of gold from various matrices can be a challenge when gold is not freely occurring and when it is associated with minerals that are difficult to penetrate through. In most cases, determining in which size screen the gold is highly recovered is important. The analysis of the various screens from the tailings sample revealed that the finer screen (-38 μm) resulted in the highest recovery of gold at 106% (Fig. 3). This can be attributed to the fact that the coarser material, which could have been a hindrance during chemical analysis, has been sieved into another fraction, therefore opening up the finer particles to attack. However, it was also seen that in the five screens that were analyzed, all of them gave an optimum recovery of gold, with recoveries starting from 72%. It was reported that the gold recovery indeed increased with decreasing particle size, with 90% < 106 μm resulting in a recovery of 93%^21^. Furthermore, at particle sizes lower than or equal to 106 μm, the percentage of particles passing through the screen did not significantly affect gold recovery. Based on these results and the literature, an ultrafine screen of -38 μm was used for further chemical analysis.

### Acid digestion

**Fig. 4:**
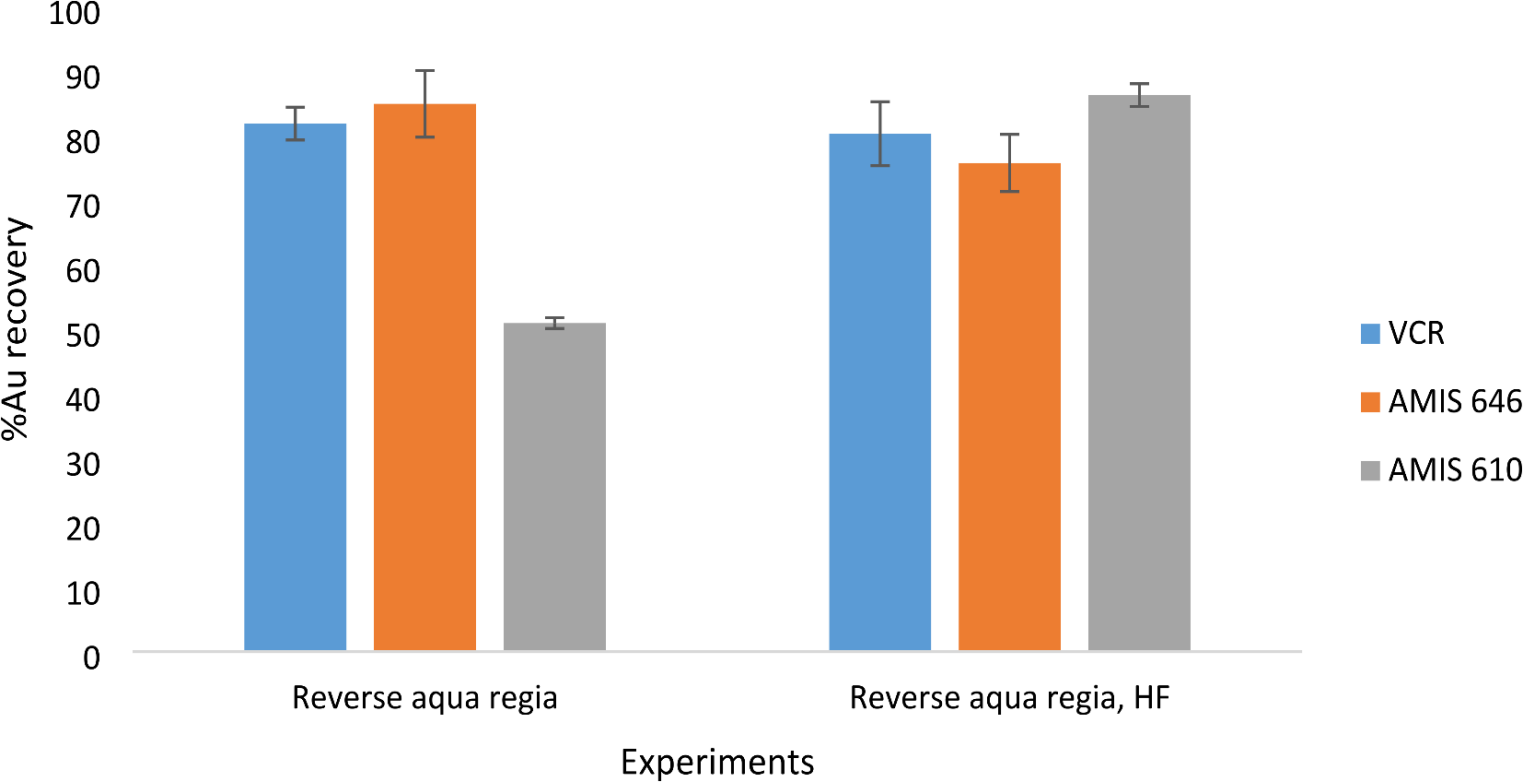
Gold recoveries obtained from the two experiments (*n* = 3). Error bars represent the standard deviation.

The main aim of this study was to identify a digestion method that can quantify gold from a tailings sample. Although various methods already exist, most of the methods use large sample masses, acid combinations that pose various safety hazards, and often require long digestion times. In this study, a simple acid mixture of HNO_3_ and HCl in a 3:1 (reverse aqua regia) ratio with and without HF was used with a sample mass of 1 g and a digestion time of 20 min. Figure 4 shows the recoveries for the 2 experiments in which HF was added. In the typical digestion of gold by aqua regia, the quantification depends on the formation of the tetrachloroaurate complex (AuCl_4_), which is dependent on the amount of chloride ions provided by the HCl, as shown in Eqs. 5 and 6^22^. When reverse aqua is used, the chloride ions are depleted, while the nitrate ions are dominant, thus leading to the formation of the tetranitroaurate complex, which is more stable than the tetrachloroaurate complex^22^.

5 $${\text{Au}}\,+\,{\text{3HN}}{{\text{O}}_{\text{3}}}\,+\,{\text{4HCl}} \to {\text{AuC}}{{\text{l}}_{\text{4}}}^{ - }~+{\text{ }}{{\text{H}}_{\text{3}}}{{\text{O}}^+}+{\text{ 3HN}}{{\text{O}}_{\text{2}}}\,+\,{\text{2}}{{\text{H}}_{\text{2}}}{\text{O,}}$$

6 $${\text{A}}{{\text{u}}^{{\text{3}}+}}+{\text{4N}}{{\text{O}}_{\text{3}}}^{ - } \to {\text{Au}}{\left( {{\text{N}}{{\text{O}}_{\text{3}}}} \right)_{\text{4}}}^{ - }.$$

The experiments yielded recoveries greater than 50% for the tailing material that had a head grade of approximately 0.30 g/t as determined by fire assay. For the VCR matrix, the concentration obtained ranged between 0.257 and 0.262 g/t, equating to recoveries of 80 to 82%, of which 82% was obtained from an experiment in which HF was not added. In most studies, HF is added in acid digestions to decompose quartz, as it hinders acid digestion, which is usually a problem when the sample mass used is large. In this study, whether HF was added or not added, the best recoveries were still obtained, indicating that the gold grains were not affected by any encapsulation. Furthermore, as per the gold depot results in Table 4, only 0.05 g.t^− 1^ is locked in quartz. This was also true for the certified reference materials that were used, which are lower in grade than the sample. Because a residue remained after digestion due to incomplete digestion, the constant addition of HNO_3_ compensated for this, as the nitrate ions were always in excess. This could also be because of the nature of the tailings, as most of the minerals are present in small quantities, and since they have been processed before, subsequent chemical analysis has become much easier.

Table 5 shows a comparison of the various acid digestion methods for gold analysis and the limits of detection (LODs) included in this study. In several studies, various LODs of 0.002 µg. L^− 1^ were obtained with analysis on GF-AAS^10^ and the same LOD as ICP‒MS^6^. Both of these methods use larger sample masses and aqua regia as the main reagents; however, they are coupled with other methods, such as solvent extraction. This means that an extra step was added after acid digestion to extract the gold into the organic phase, which often requires special instrumental compartments. Based on the results of this study, acid digestion was sufficient to extract the majority of the gold and analyze the ICP‒MS data. This is supported by the limits of detection, as shown in Table 5, which ranged between 0.139 and 0.183 µg.kg^− 1^.

**Table 5:** Comparison of the proposed methods to published methods for the quantification of gold from geological samples, including tailings.

Method	Key reagents	Sample mass, g	Analysis method	LOD, µg.kg^− 1^	Reference
Acid digestion with solvent extraction	HNO_3_ + HF + HCl DLLME	10	GF-AAS	0.0020	^10^
Acid digestion	AR	10	GF-AAS	0.23	^9^
Acid digestion	HNO_3_ + HF + HCl + AR DIBK-loaded CG71 resin	4	ICP‒MS	0.0020	^6^
Acid digestion	Reverse aqua regia	1	ICP‒MS	-	^22^
Acid digestion	Reverse aqua regia	1	ICP‒MS	0.18	This study
Acid digestion	Reverse aqua regia with HF	1	ICP‒MS	0.14	This study

### Statistical evaluations

Uncertainty studies were carried out to compare the acid digestion method with reverse aqua regia without the addition of HF to the conventional fire assay method. In Table 6, the quantified uncertainty contributors are shown together with the combined and expanded uncertainties. For Type B uncertainty, the standard uncertainty was determined by taking the quotient of the expanded uncertainty on the certificate and the coverage factor. For Type A uncertainty, the standard deviation was taken as the standard uncertainty for repetitive measurements such as for homogeneity and repeatability (precision).

### Uncertainty due to weighing

The calibration certificate reported an expanded uncertainty of 0.0003 g at a coverage factor of 2; therefore, the uncertainty due to weighing of the sample is given by:

$$\:{u}_{s}=\frac{Expanded\:uncertainty}{coverage\:factor}=\frac{0.0003}{2}=0.00015$$

### Uncertainty due to the use of volumetric glassware

A 50 mL volumetric flask was used (at 23 °C), which has an uncertainty of 0.05 mL at 20 °C and follows a triangular distribution ($\:k=\sqrt{3}$). The uncertainty is then given by:

$$\:{u}_{1}=\frac{0.05}{\sqrt{3}}=0.02041\:$$

Due to the temperature variation, the uncertainty contributed by the variation is calculated as:

$$\:{u}_{2}=\frac{volume\times\:expansion\:factor\times\:temperature\:difference}{coverage\:factor}$$

$$\:{u}_{2}=\frac{50\times\:(2.1\times\:1{0}^{-4})\times\:3}{1.96}$$

$$\:{u}_{2}=0.160714$$

The combined uncertainty contributed by the use of volumetric glassware:

$$\:{u}_{glassware}=\sqrt{{\left(0.02041\right)}^{2}+{\left(0.160714\right)}^{2}}=0.16$$

$$\:{u}_{glassware}relative=\frac{0.16}{50}=0.0032$$

### Uncertainty of Dilution of the working standard

A working standard of 1 µg.mL^− 1^ was prepared from a 1000 µg.mL^− 1^ stock solution, with an uncertainty of 5 µg.mL^− 1^, which follows a rectangular distribution (k=$\:\sqrt{3})$. The uncertainty due to dilution of the stock solution is:

$$\:{u}_{stock}=\frac{5}{\sqrt{3}}=2.88675$$

The working standard was diluted to make calibration standards in the range of 0.005–0.1 µg.mL^− 1^. Repetitive dilutions of the lower and upper standards gave standard deviations of 0.000387 (u_v1_) and 0.000936 mL (u_v2_), respectively. The overall uncertainty as a result of the dilution of the working standard is calculated by:

$$\:{u}_{working\:standar}relative=\sqrt{{\left(\frac{{u}_{stock}}{\left[stock\right]}\right)}^{2}+{\left(\frac{{u}_{v1}}{dispensed\:volume}\right)}^{2}+{\left(\frac{{u}_{v2}}{dispensed\:volume}\right)}^{2}}$$

$$\:{u}_{working\:standard}relative=\sqrt{{\left(\frac{2.88675}{1000}\right)}^{2}+{\left(\frac{0.000387}{0.5}\right)}^{2}+{\left(\frac{0.000936}{5}\right)}^{2}}$$

$$\:{u}_{working\:standard}relative=0.5\times\:3.\:44\times\:{10}^{-3}=0.0017215\:$$

### Uncertainty due to dilution factor

A dilution factor of 5 was used for all the acid digestion samples with a final volume of 10 mL; therefore, a volume of 2 mL was taken from each sample. The standard deviations associated with the repetitive dispensing of 2 mL and 10 mL were calculated as 0.000237 and 0.000103 mL, respectively. These standard deviations are then used in the equation below to calculate the uncertainty arising from the dilution factor.

$$\:=5\times\:\sqrt{{\left(\frac{0.000237}{2}\right)}^{2}+{\left(\frac{0.000103}{10}\right)}^{2}}$$

$$\:{u}_{dilution\:factor}relative=0.0001189$$

According to the data obtained (Table 6), the uncertainties between the two techniques are comparable. The major contributors to the uncertainty are the method repeatability (0.031 and 0.038 g.t^− 1^) and reproducibility (0.014 and 0.0093 g.t^− 1^) for both techniques. This was expected because although the fire assay is good for the quantification of gold, it does suffer from minimal repeatability issues, which is due to the nature of the process. Homogeneity contributes more to errors in chemical analysis, especially when there are nugget effects to consider^8^. This experiment and calculations contributed approximately 12% of the overall uncertainty. This is satisfactory considering that a sample mass of 1 g was used in the acid digestion.

**Table 6:** Quantification of the uncertainties from various parameters from acid digestion and fire assays with an ICP‒MS finish.

Contributor	Acid digestion	Fire assay
Mass balance	0.00015	0.00015
Glassware calibration	0.0032	0.0032
Homogeneity	0.031	0.034
Repeatability	0.031	0.038
Reproducibility	0.014	0.0093
Instrument stability	0.00038	0.00054
Concentration of dilute working standard	0.0017	0.0017
Dilution factor	0.00012	0.00012
Combined uncertainty	0.046	0.052
Expanded uncertainty	0.092	0.10
Quantified value and uncertainty	0.258 ± 0.092 g/t	0.26 ± 0.10 g/t

The data in Table 6 are further supported by a paired t test, which showed that when comparing the means of the two techniques, there is no significant difference, with a t_statistic_ calculated as -1.59 and a t_critical_ calculated as 0.15. Subsequently, the challenge is, if both methods result in similar uncertainties, which method is then chosen and used. Based on the method’s level of difficulty, less time needed, and lack of use of toxic reagents such as lead, acid digestion is the best recommended, and it does not require intensive skills compared to fire assay.

## Conclusion

The purpose of the present study was to assess the suitability of acid digestion using reverse aqua regia for the quantification of gold from mine tailings and to compare its uncertainty with that obtained by fire assay. The study revealed that by using a finer screen (-38 μm) in terms of particle size, satisfactory homogeneity is obtained, which means that in terms of using a sample mass of 1 g, the analysis should not be affected by homogeneity. Furthermore, good gold recoveries of 82% were obtained regardless of the presence or absence of HF, implying that encapsulation of the gold grains by minerals such as quartz was not a hindrance. The uncertainty evaluation ranged between 0.092 and 0.10 g.t^− 1^ for the acid digestion and fire assays, respectively. This was impressive, as it implies that the acid digestion method is comparable to the fire assay. Overall, the reverse aqua regia method, which is used on a fine screen, showed great performance and can be used successfully with low uncertainties for the quantification of gold in mine tailings.

## Data Availability

The raw data used to support the findings of the study will be made available to anyone who requests it by the corresponding author.

## Abbreviations

ANOVA: Analysis of variance
AR: Aqua regia
AuCl_4_: Tetrachloroaurate
CIL: Carbon in leach
CRM: Certified reference material
DLLME: Dispersive liquid‒liquid microextraction
FA-AAS: Fire assay with atomic absorption spectroscopy
FA-ICP‒MS: Fire assay with induced coupled plasma mass spectrometry
FA-ICP‒OES: Fire assay with induced coupled plasma optical emission spectroscopy
GF-AAS: Graphite furnace with atomic absorption spectroscopy
H_2_O_2_: Hydrogen peroxide
HCl: Hydrochloric acid
HCLO_4_: Perchloric acid
HF: Hydrofluoric acid
HNO_3_: Nitric acid
INAA: Instrumental neuron activation analysis
LOD: Limit of detection
Na_2_CO_3_: Sodium carbonate
Na_2_O_2_: Sodium peroxide
NaCN: Sodium cyanide
PSD: Particle size distribution
RSD: Relative standard deviation
SiF_4_: Silicon tetrafluoride
UNC: Uncertainty
XRD: X-ray diffraction
